# Supplementary material for: Gene mapping methodology powered by induced genome rearrangements
Source: Sci Rep. 2022 Oct 5;12:16658. doi: 10.1038/s41598-022-20999-7 (PMC9534892; doi:10.1038/s41598-022-20999-7)
Supplement: Supplementary file 1 — Supplementary Information. [file 41598_2022_20999_MOESM1_ESM.pdf]

Gene mapping methodology powered by induced genome rearrangements

Hideyuki Yone[1], Hiromitsu Kono[1], Hayato Hirai[1], Kunihiro Ohta[1,2,*]

[1]Department of Life Sciences, Graduate School of Arts and Sciences,
The University of Tokyo, Komaba 3-8-1, Meguro-ku, Tokyo 153-8902, Japan
[2]Universal Biology Institute, The University of Tokyo,
Hongo 7-3-1, Bunkyo-Ku, Tokyo 113-0033, Japan.

*Corresponding author: K. Ohta (kohta-pub2[at]bio.c.u-tokyo.ac.jp)
Tel & Fax, +81-3-5465-8834

Supplementary Information: Figure 1-5, and Table 1.

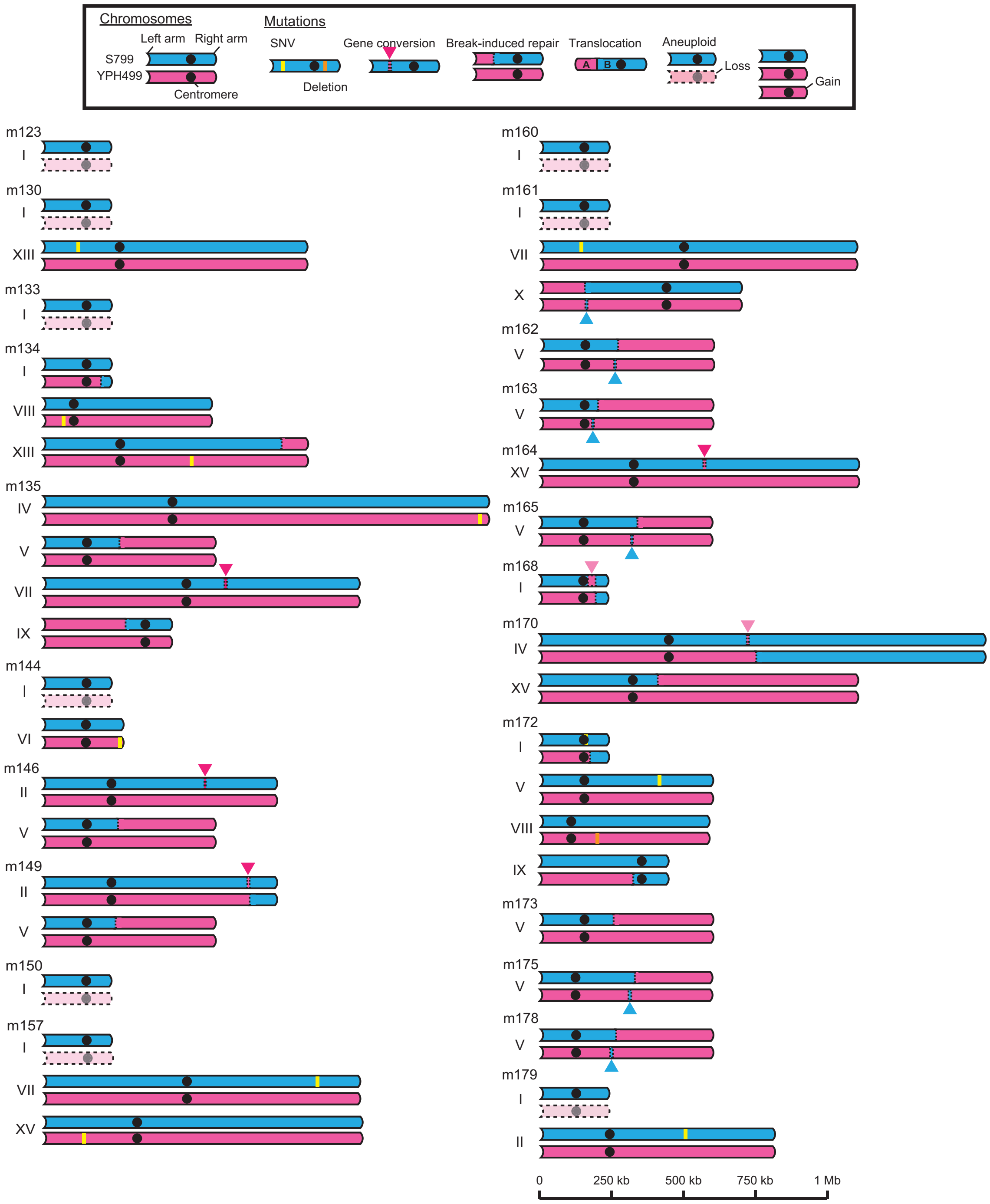

Supplementary Fig. 1. Rearranged chromosomes in TAQed mutants. Schematic diagrams of rearranged chromosomes in non-flocculent TAQed mutants m123, m130, m133, m134, m135, m144, m146, m149, m150, m157, m160, m161, m162, m163, m164, m165, m168, m170, m172, m173, m175, m178, and m179. S799 chromosomes, blue; YPH499 chromosomes, magenta.

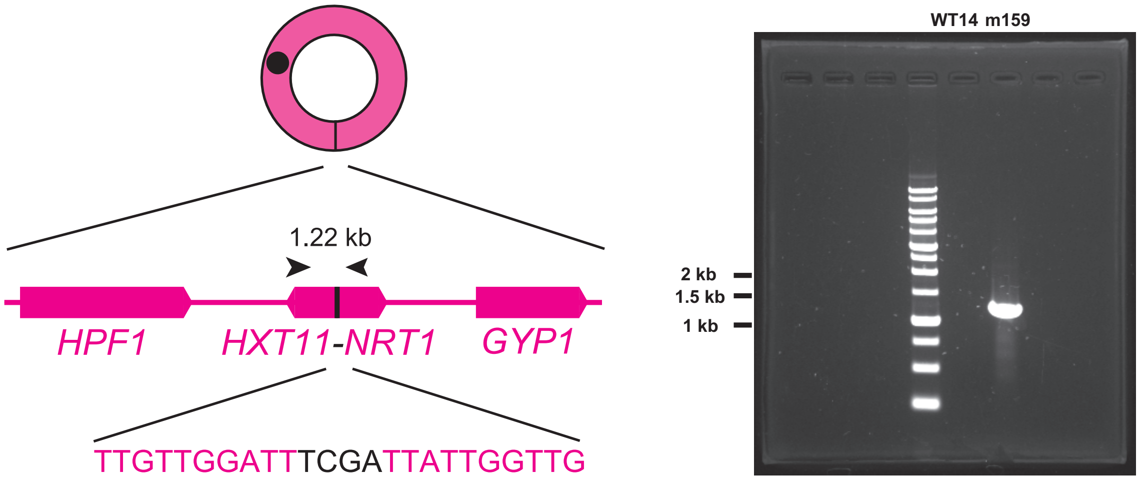

Supplementary Fig. 2. Breakpoint sequence of presumed circular chromosome in m159. Breakpoints are at TaqI-recognition sequences (5′-TCGA-3′) located within *HXT11* and *NRT1* gene in YPH499-derived chromosome XV. Presumed circularization is verified by polymerase chain reaction (PCR). PCR primers are indicated by black arrows. An uncropped electrophoresis image is shown.

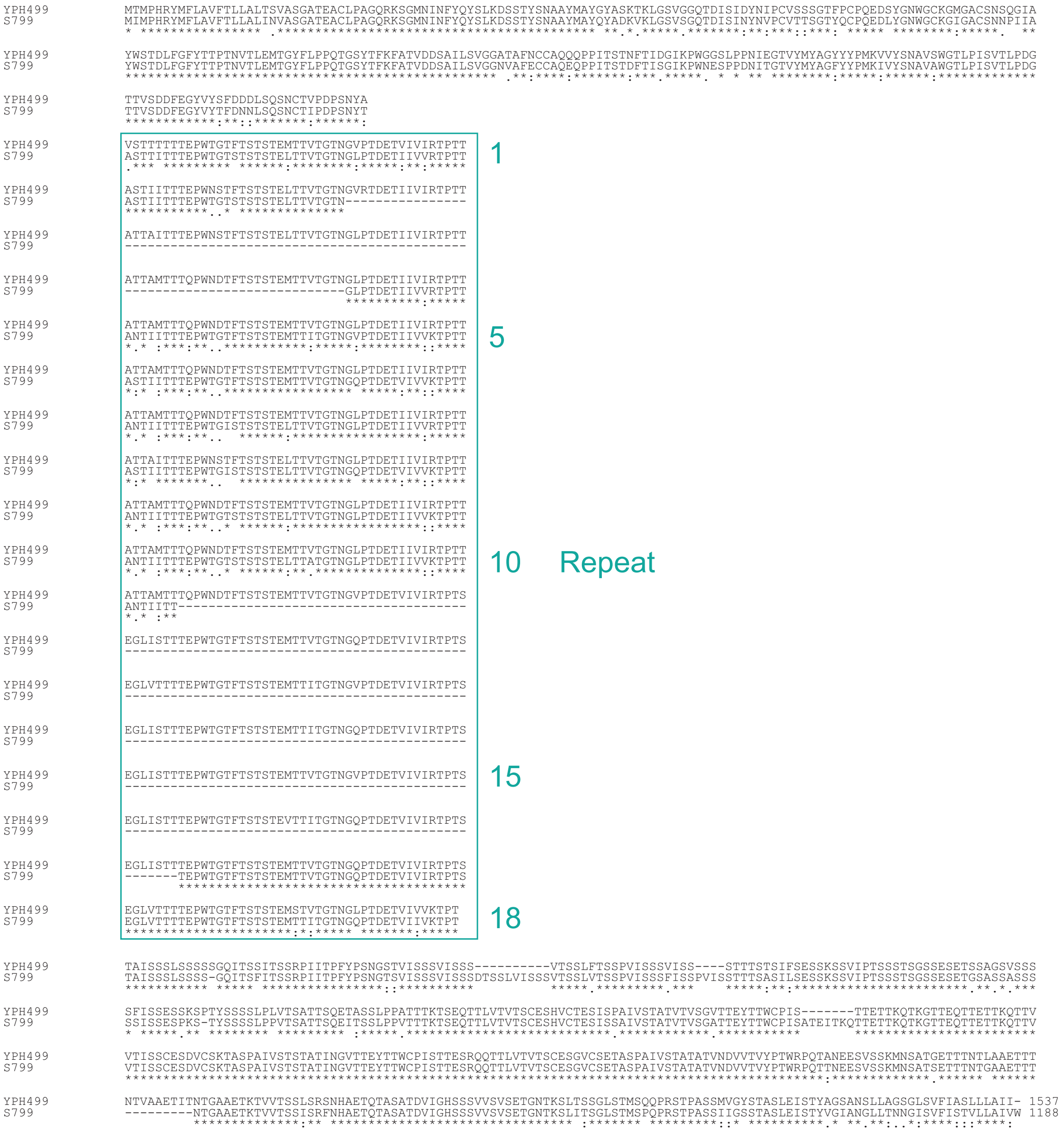

Supplementary Fig. 3. Amino acid sequence alignment of Flo1 protein between YPH499 (upper) and S799 (bottom).

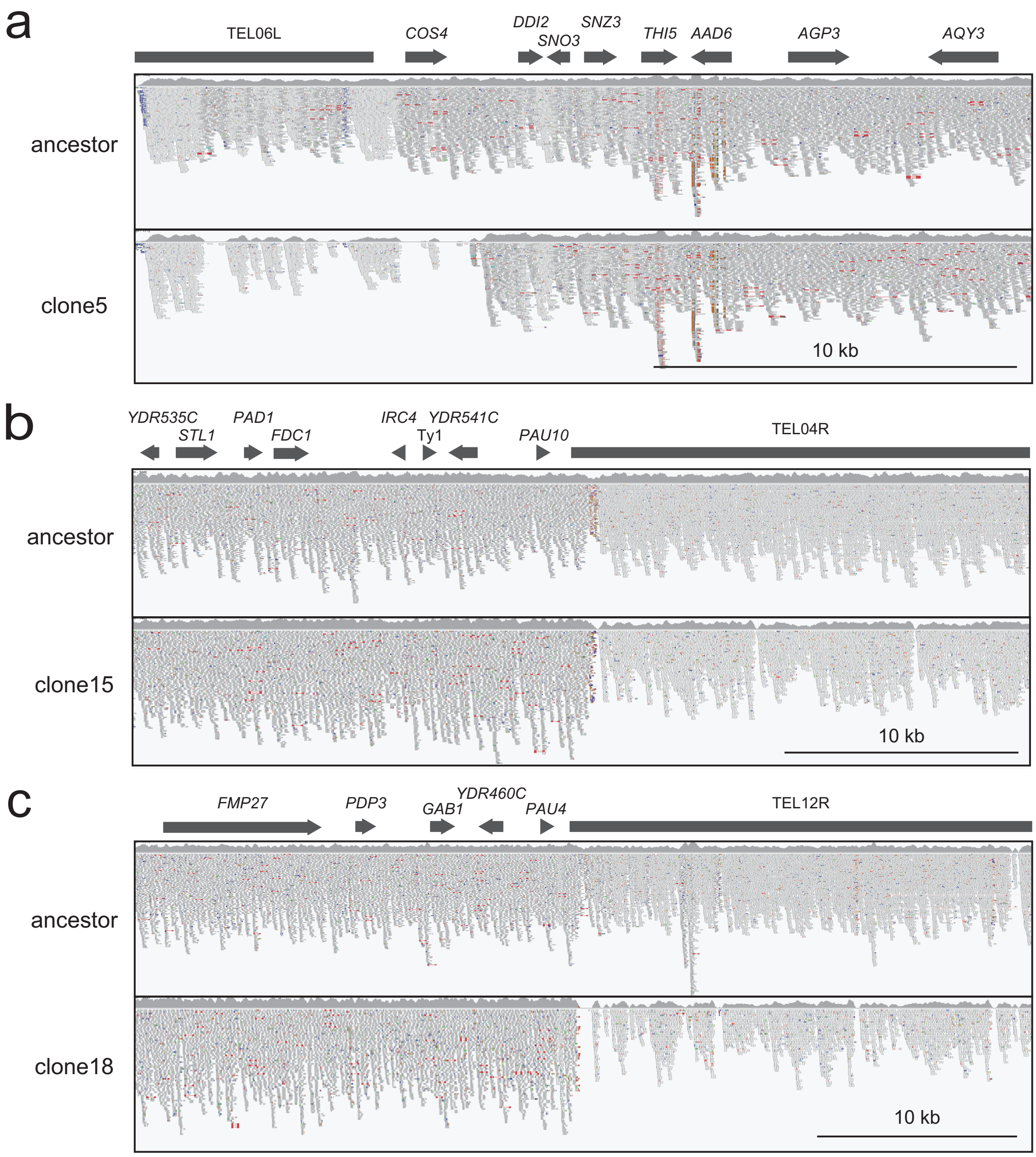

Supplementary Fig. 4. Telomeric deletion in experimentally evolved clones. Sequence-read alignments for the telomeric regions of the (a) left arm of chromosome VI in clone 5, (b) right arm of chromosome IV in clone 15, and (c) right arm of chromosome XII in clone 18 are visualized by IGV.

```
clone1          MTMPHRYMFLAVFTLLALTSVASGATEACLPAGQRKSGMNINFYQYSLKDSSTYSNAAYMAYGYASKTKLGSVGGQTDISIDYNIPCVSSSGTFPCPQEDSYGNWGCKGMGACSNSQGIA
YPH499          MTMPHRYMFLAVFTLLALTSVASGATEACLPAGQRKSGMNINFYQYSLKDSSTYSNAAYMAYGYASKTKLGSVGGQTDISIDYNIPCVSSSGTFPCPQEDSYGNWGCKGMGACSNSQGIA
                ************************************************************************************************************************

clone1          YWSTDLFGFYTTPTNVTLEMTGYFLPPQTGSYTFKFATVDDSAILSVGGATAFNCCAQQQPPITSTNFTIDGIKPWGGSLPPNIEGTVYMYAGYYYPMKVVYSNAVSWGTLPISVTLPDG
YPH499          YWSTDLFGFYTTPTNVTLEMTGYFLPPQTGSYTFKFATVDDSAILSVGGATAFNCCAQQQPPITSTNFTIDGIKPWGGSLPPNIEGTVYMYAGYYYPMKVVYSNAVSWGTLPISVTLPDG
                ************************************************************************************************************************

clone1          TTVSDDFEGYVYSFDDDLSQSNCTVPDPSNYA
YPH499          TTVSDDFEGYVYSFDDDLSQSNCTVPDPSNYA
                ********************************

clone1          VSTTTTTTEPWTGTFTSTSTEMTTVTGTN----------------
YPH499          VSTTTTTTEPWTGTFTSTSTEMTTVTGTNGVPTDETVIVIRTPTT   1   Repeat
                *****************************

clone1          ---------------------------------------------
YPH499          ASTIITTTEPWNSTFTSTSTELTTVTGTNGVRTDETIIVIRTPTT

clone1          ---------------------------------------------
YPH499          ATTAITTTEPWNSTFTSTSTELTTVTGTNGLPTDETIIVIRTPTT

clone1          ---------------------------------------------
YPH499          ATTAMTTTQPWNDTFTSTSTEMTTVTGTNGLPTDETIIVIRTPTT

clone1          ---------------------------------------------
YPH499          ATTAMTTTQPWNDTFTSTSTEMTTVTGTNGLPTDETIIVIRTPTT   5

clone1          ---------------------------------------------
YPH499          ATTAMTTTQPWNDTFTSTSTEMTTVTGTNGLPTDETIIVIRTPTT

clone1          ---------------------------------------------
YPH499          ATTAMTTTQPWNDTFTSTSTEMTTVTGTNGLPTDETIIVIRTPTT

clone1          ---------------------------------------------
YPH499          ATTAITTTEPWNSTFTSTSTELTTVTGTNGLPTDETIIVIRTPTT

clone1          ---------------------------------------------
YPH499          ATTAMTTTQPWNDTFTSTSTEMTTVTGTNGLPTDETIIVIRTPTT

clone1          ---------------------------------------------
YPH499          ATTAMTTTQPWNDTFTSTSTEMTTVTGTNGLPTDETIIVIRTPTT   10

clone1          -----------------------------GVPTDETVIVIRTPTS
YPH499          ATTAMTTTQPWNDTFTSTSTEMTTVTGTNGVPTDETVIVIRTPTS
                                             ****************

clone1          EGLISTTTEPWTGTFTSTSTEMTTVTGTNGQPTDETVIVIRTPTS
YPH499          EGLISTTTEPWTGTFTSTSTEMTTVTGTNGQPTDETVIVIRTPTS
                *********************************************

clone1          EGLVTTTTEPWTGTFTSTSTEMTTITGTNGVPTDETVIVIRTPTS
YPH499          EGLVTTTTEPWTGTFTSTSTEMTTITGTNGVPTDETVIVIRTPTS
                *********************************************

clone1          EGLISTTTEPWTGTFTSTSTEMTTITGTNGQPTDETVIVIRTPTS
YPH499          EGLISTTTEPWTGTFTSTSTEMTTITGTNGQPTDETVIVIRTPTS
                *********************************************

clone1          EGLISTTTEPWTGTFTSTSTEMTTVTGTNGVPTDETVIVIRTPTS
YPH499          EGLISTTTEPWTGTFTSTSTEMTTVTGTNGVPTDETVIVIRTPTS   15
                *********************************************

clone1          EGLISTTTEPWTGTFTSTSTEVTTITGTNGQPTDETVIVIRTPTS
YPH499          EGLISTTTEPWTGTFTSTSTEVTTITGTNGQPTDETVIVIRTPTS
                *********************************************

clone1          EGLISTTTEPWTGTFTSTSTEMTTVTGTNGQPTDETVIVIRTPTS
YPH499          EGLISTTTEPWTGTFTSTSTEMTTVTGTNGQPTDETVIVIRTPTS
                *********************************************

clone1          EGLVTTTTEPWTGTFTSTSTEMSTVTGTNGLPTDETVIVVKTPTT
YPH499          EGLVTTTTEPWTGTFTSTSTEMSTVTGTNGLPTDETVIVVKTPTT   18
                *********************************************

clone1          AISSSLSSSSSGQITSSITSSRPIITPFYPSNGTSVISSSVISSSVTSSLFTSSPVISSSVISSSTTTSTSIFSESSKSSVIPTSSSTSGSSESETSSAGSVSSSSFISSESSKSPTYSS
YPH499          AISSSLSSSSSGQITSSITSSRPIITPFYPSNGTSVISSSVISSSVTSSLFTSSPVISSSVISSSTTTSTSIFSESSKSSVIPTSSSTSGSSESETSSAGSVSSSSFISSESSKSPTYSS
                ************************************************************************************************************************

clone1          SSLPLVTSATTSQETASSLPPATTTKTSEQTTLVTVTSCESHVCTESISPAIVSTATVTVSGVTTEYTTWCPISTTETTKQTKGTTEQTTETTKQTTVVTISSCESDVCSKTASPAIVST
YPH499          SSLPLVTSATTSQETASSLPPATTTKTSEQTTLVTVTSCESHVCTESISPAIVSTATVTVSGVTTEYTTWCPISTTETTKQTKGTTEQTTETTKQTTVVTISSCESDVCSKTASPAIVST
                ************************************************************************************************************************

clone1          STATINGVTTEYTTWCPISTTESRQQTTLVTVTSCESGVCSETASPAIVSTATATVNDVVTVYPTWRPQTANEESVSSKMNSATGETTTNTLAAETTTNTVAAETITNTGAAETKTVVTS
YPH499          STATINGVTTEYTTWCPISTTESRQQTTLVTVTSCESGVCSETASPAIVSTATATVNDVVTVYPTWRPQTANEESVSSKMNSATGETTTNTLAAETTTNTVAAETITNTGAAETKTVVTS
                ************************************************************************************************************************

clone1          SLSRSNHAETQTASATDVIGHSSSVVSVSETGNTKSLTSSGLSTMSQQPRSTPASSMVGYSTASLEISTYAGSANSLLAGSGLSVFIASLLLAII  1087
YPH499          SLSRSNHAETQTASATDVIGHSSSVVSVSETGNTKSLTSSGLSTMSQQPRSTPASSMVGYSTASLEISTYAGSANSLLAGSGLSVFIASLLLAII  1537
                ***********************************************************************************************
```

Supplementary Fig. 5. Amino acid sequence alignment of Flo1 protein between experimentally evolved clone 1 (upper) and YPH499 (bottom).

Supplementary Table 1-1. Structural variants of TAQed strains.

Strain	Rearrange type	Chromosome	Position	Mutation
m126	SNV	YPH499chr08	138,529	C > G
m130	SNV	S799chr13	123,861	G > A
m131	SNV	YPH499chr08	147,194	C > A
m131	SNV	S799chr15	64,350	A > G
m134	SNV	YPH499chr08	70,964	A > G
m134	SNV	YPH499chr13	516,844	A > C
m135	SNV	YPH499chr04	1,517,959	T > C
m144	SNV	YPH499chr06	271,238	G > T
m157	SNV	YPH499chr15	138,794	T > C
m157	SNV	S799chr07	950,059	G > T
m159	SNV	S799chr12	840,323	G > A
m161	SNV	S799chr07	142,504	A > G
m166	SNV	S799chr13	585,981	C > T
m172	SNV	S799chr01	158,942	T > C
m172	SNV	S799chr05	413,007	G > A
m174	SNV	YPH499chr07	17,393	T > G
m177	SNV	S799chr09	286,867	T > G
m179	SNV	S799chr02	503,926	G > A
Strain	Rearrange type	Chromosome	Start	End
m126	SGC	YPH499chr09	387,500	390,900
m126	SGC	YPH499chr16	165,500	167,700
m131	SGC	YPH499chr08	140,500	146,400
m135	SGC	S799chr07	624,900	632,000
m146	SGC	S799chr02	561,900	562,900
m149	SGC	S799chr02	710,200	718,500
m159	SGC	YPH499chr07	959,400	965,000
m161	SGC	YPH499chr10	151,800	161,900
m162	SGC	YPH499chr05	252,600	255,000
m163	SGC	YPH499chr05	177,400	187,000
m164	SGC	S799chr15	565,802	570,600
m165	SGC	YPH499chr05	315,805	326,000
m168	SGC	YPH499chr01	164,100	194,300
m169	SGC	YPH499chr01	197,300	208,100
m170	SGC	S799chr04	728,500	729,200
m174	SGC	YPH499chr04	1,033,100	1,037,800
m174	SGC	YPH499chr07	17,500	30,700
m174	SGC	YPH499chr13	283,700	293,600
m174	SGC	YPH499chr13	301,400	303,300
m175	SGC	YPH499chr05	308,300	318,600
m177	SGC	YPH499chr15	519,100	521,100
m178	SGC	YPH499chr05	243,100	251,000

Blue data represent the approximate rearrangement regions, whereas black data represent precise positions. (SNV; Single Nucleotide Variation, SGC; Short Gene Conversion)

Supplementary Table 1-2. Structural variants of TAQed strains.

Strain	Rearrange type	Left arm	Right arm	Rearranged position
m131	BIR	S799chr16	YPH499chr16	46,600
m134	BIR	YPH499chr01	S799chr01	204,400
m134	BIR	S799chr13	YPH499chr13	829,000
m135	BIR	S799chr05	YPH499chr05	272,200
m135	BIR	YPH499chr09	S799chr09	287,000
m146	BIR	S799chr05	YPH499chr05	263,900
m149	BIR	YPH499chr02	S799chr02	719,800
m149	BIR	S799chr05	YPH499chr05	251,500
m159	BIR	S799chr07	YPH499chr07	234,700
m159	BIR	S799chr05	YPH499chr05	356,500
m161	BIR	YPH499chr10	S799chr10	151,800
m162	BIR	S799chr05	YPH499chr05	266,700
m163	BIR	S799chr05	YPH499chr05	199,600
m165	BIR	S799chr05	YPH499chr05	339,300
m166	BIR	YPH499chr05	S799chr05	260,400
m168	BIR	YPH499chr01	S799chr01	194,300
m170	BIR	YPH499chr04	S799chr04	752,400
m170	BIR	S799chr15	YPH499chr15	410,800
m172	BIR	YPH499chr01	S799chr01	172,600
m172	BIR	YPH499chr09	S799chr09	326,400
m173	BIR	S799chr05	YPH499chr05	253,000
m174	BIR	S799chr05	YPH499chr05	319,500
m175	BIR	S799chr05	YPH499chr05	330,300
m177	BIR	S799chr15	YPH499chr15	500,000
m178	BIR	S799chr05	YPH499chr05	263,600

Strain	Rearrange type	Chromosome	Alteration
m123	Aneuploidy	YPH499chr01	Loss
m126	Aneuploidy	YPH499chr01	Loss
m130	Aneuploidy	YPH499chr01	Loss
m133	Aneuploidy	YPH499chr01	Loss
m144	Aneuploidy	YPH499chr01	Loss
m150	Aneuploidy	YPH499chr01	Loss
m157	Aneuploidy	YPH499chr01	Loss
m159	Aneuploidy	YPH499chr11	Loss
m160	Aneuploidy	YPH499chr01	Loss
m161	Aneuploidy	YPH499chr01	Loss
m166	Aneuploidy	YPH499chr01	Loss
m166	Aneuploidy	YPH499chr12	Gain
m179	Aneuploidy	YPH499chr01	Loss

Strain	Rearrange type	Chromosome	Region	Chromosome	Region
m131	TL	YPH499chr01	161,900 (Ty element)	YPH499chr16	56,900 (Ty element)
m159	Circularization	YPH499chr15	26,353	YPH499chr15	466,447
m172	Deletion	YPH499chr08	193,991	YPH499chr08	195,216
m177	TL	YPH499chr01	161,900 (Ty element)	S799chr03	178,000 (Ty element)

Blue data represent the approximate rearrangement regions, whereas black data represent precise positions. (BIR; Break-induced repair, TL; Translocation)
